# Deep Learning-Based Classification of Cancer Cell in Leptomeningeal Metastasis on Cytomorphologic Features of Cerebrospinal Fluid

**DOI:** 10.3389/fonc.2022.821594

**Published:** 2022-02-22

**Authors:** Wenjin Yu, Yangyang Liu, Yunsong Zhao, Haofan Huang, Jiahao Liu, Xiaofeng Yao, Jingwen Li, Zhen Xie, Luyue Jiang, Heping Wu, Xinhao Cao, Jiaming Zhou, Yuting Guo, Gaoyang Li, Matthew Xinhu Ren, Yi Quan, Tingmin Mu, Guillermo Ayuso Izquierdo, Guoxun Zhang, Runze Zhao, Di Zhao, Jiangyun Yan, Haijun Zhang, Junchao Lv, Qian Yao, Yan Duan, Huimin Zhou, Tingting Liu, Ying He, Ting Bian, Wen Dai, Jiahui Huai, Xiyuan Wang, Qian He, Yi Gao, Wei Ren, Gang Niu, Gang Zhao

**Affiliations:** ^1^ Department of Neurology, Xijing Hospital, the Fourth Military Medical University, Xi’an, China; ^2^ Department of Neurology, Yan’an University Medical College No. 3 Affiliated Hospital, Xianyang, China; ^3^ The College of Life Sciences and Medicine, Northwest University, Xi’an, China; ^4^ Electronic Materials Research Laboratory, Key Laboratory of the Ministry of Education & International Center for Dielectric Research, School of Electronic Science and Engineering & The International Joint Laboratory for Micro/Nano Manufacturing and Measurement Technology, Xi’an Jiaotong University, Xi’an, China; ^5^ School of Biomedical Engineering, Health Science Center, Shenzhen University, Shenzhen, China; ^6^ The College of Medicine, Xiamen University, Xiamen, China; ^7^ Ophthalmology, Department of Clinical Science, Lund University, Lund, Sweden; ^8^ Institute of Fluid Science, Tohoku University, Sendai, Japan; ^9^ Biology Program, Faculty of Science, The University of British Columbia, Vancouver, BC, Canada; ^10^ School of Microelectronics, Xidian University, Xi’an, China; ^11^ Multiple Sclerosis Unit, Neurology Service, Vithas Nisa Hospital, Seville, Spain; ^12^ Department of Ophthalmology, Eye Institute of PLA, Xijing Hospital, Fourth Military Medical University, Xi’an, China; ^13^ Department of Neurology, Xiji Country People’s Hospital, Ningxia, China; ^14^ Shenzhen Key Laboratory of Precision Medicine for Hematological Malignancies, Shenzhen, Guangzhou, China; ^15^ Marshall Laboratory of Biomedical Engineering, Shenzhen, China; ^16^ Peng Cheng Laboratory, Shenzhen, China

**Keywords:** leptomeningeal metastasis (LM), deep learning, cytology, CSF, cancer cell

## Abstract

**Background:**

It is a critical challenge to diagnose leptomeningeal metastasis (LM), given its technical difficulty and the lack of typical symptoms. The existing gold standard of diagnosing LM is to use positive cerebrospinal fluid (CSF) cytology, which consumes significantly more time to classify cells under a microscope.

**Objective:**

This study aims to establish a deep learning model to classify cancer cells in CSF, thus facilitating doctors to achieve an accurate and fast diagnosis of LM in an early stage.

**Method:**

The cerebrospinal fluid laboratory of Xijing Hospital provides 53,255 cells from 90 LM patients in the research. We used two deep convolutional neural networks (CNN) models to classify cells in the CSF. A five-way cell classification model (CNN1) consists of lymphocytes, monocytes, neutrophils, erythrocytes, and cancer cells. A four-way cancer cell classification model (CNN2) consists of lung cancer cells, gastric cancer cells, breast cancer cells, and pancreatic cancer cells. Here, the CNN models were constructed by Resnet-inception-V2. We evaluated the performance of the proposed models on two external datasets and compared them with the results from 42 doctors of various levels of experience in the human-machine tests. Furthermore, we develop a computer-aided diagnosis (CAD) software to generate cytology diagnosis reports in the research rapidly.

**Results:**

With respect to the validation set, the mean average precision (mAP) of CNN1 is over 95% and that of CNN2 is close to 80%. Hence, the proposed deep learning model effectively classifies cells in CSF to facilitate the screening of cancer cells. In the human-machine tests, the accuracy of CNN1 is similar to the results from experts, with higher accuracy than doctors in other levels. Moreover, the overall accuracy of CNN2 is 10% higher than that of experts, with a time consumption of only one-third of that consumed by an expert. Using the CAD software saves 90% working time of cytologists.

**Conclusion:**

A deep learning method has been developed to assist the LM diagnosis with high accuracy and low time consumption effectively. Thanks to labeled data and step-by-step training, our proposed method can successfully classify cancer cells in the CSF to assist LM diagnosis early. In addition, this unique research can predict cancer’s primary source of LM, which relies on cytomorphologic features without immunohistochemistry. Our results show that deep learning can be widely used in medical images to classify cerebrospinal fluid cells. For complex cancer classification tasks, the accuracy of the proposed method is significantly higher than that of specialist doctors, and its performance is better than that of junior doctors and interns. The application of CNNs and CAD software may ultimately aid in expediting the diagnosis and overcoming the shortage of experienced cytologists, thereby facilitating earlier treatment and improving the prognosis of LM.

## Introduction

Leptomeningeal metastasis (LM) is defined as invading the cancer cell to the leptomeninges (pia and arachnoid) surrounding the brain and the spinal cord. Approximately 5% of patients with advanced cancer are diagnosed with LM (1, 2), and this is also detected in up to 20% of cases in autopsy studies (3). LM originates from breast cancer, lung cancer, melanoma, gastrointestinal cancer, and unknown primary cancer cells (1, 2, 4), and meningeal is less commonly associated with brain tumors and hematological malignancies. The prognosis of LM is relatively poor, where the median survival ranges from 4 to 6 weeks if treatment is not received. Efficient treatment can prolong the survival time to about 2 or 3 months (5, 6). Current treatment strategies include radiotherapy, systemic antineoplastic agents, and intrathecal chemotherapy. Therefore, an early diagnosis of LM can bring a precious time for the treatment of patients.

However, challenges such as hidden onset and diversified clinical manifestations of LM increase the difficulty of diagnosing the disease in the early stage. Lumbar puncture, one of the essential tools (over 90% sensitivity) to assist central nervous system (CNS) diagnosis involvement by the cancer cell, is routinely performed on patients if no contraindications are present. When cytology is negative and the rest of the diagnostic evaluation indicators are positive, LM symptoms should also perform LM diagnosis (7). Usually, cytologists interpret cytomorphologic features of cells in cerebrospinal fluid (CSF) and spend two working days in the normal process of cytology report. A cytologist must accumulate several years of working experience to diagnose cancer cells.

Nevertheless, a cerebrospinal fluid examination lab shortage restricts getting the report promptly, delaying the patient diagnosis. Another limitation is that the entire process of generating CSF cytology reports is time-consuming, labor-intensive, complicated, and reproducible work. Quantitative and repetitive work may increase the rate of misclassified cells in the nervous system, resulting in false disease diagnosis such as missed or misdiagnosed of LM or improper treatment.

Artificial intelligence (AI) can revolutionize disease diagnosis and management by reviewing immense amounts of images promptly and solving complex classification tasks of human experts. Deep-learning-based methods have been successfully implemented in many clinical diagnoses (8–12). Compared with traditional machine learning methods, deep learning (DL) can produce a more reasonable output for the test dataset by using multiple processing layers to learn internal data representations from the training dataset (13). However, the current accuracy of the initial application of deep learning in clinical diagnosis is relatively low. Thus, a more generalized model is needed to improve the accuracy. A generic object detection neural network like a region-based convolutional neural network (R-CNN) was explored and found to be more effective in the classification task (14). ResNet CNN, raised by Xie et al. (15), was used to classify white blood cells in peripheral blood smears (16). Sahlol et al. presented a CNN based on a statistically enhanced Salp Swarm Algorithm to classify the bone marrow (BM) cells into two types (17). To date, there are few deep learning studies on cells in CSF.

So far, the cytomorphologic spectrum of findings in CSF involved by subtype of cancer cell has not been well studied. AI advancement is promising to apply AI techniques for auxiliary diagnosis of cancer cells in CSF. This study used fast-RCNN to propose an efficient and fully automated hierarchical deep learning framework for cerebrospinal fluid cytology, applied to the CNN model. The proposed framework can identify cell trajectories and cell types. Fast-RCNN has the following capabilities: (i) rapid positioning of cell trajectories and (ii) CSF cell detection and classification. This study aims to characterize the cytomorphologic spectrum of CSF involvement by LM. In particular, we propose to use CNN models to extract the features of cells in CSF based on former research to accurately identify and classify cancer cells in CSF. In addition, we build a CAD system to transfer CNN output results to a visual CSF cytology report in a few seconds, aiming to increase cytologists’ efficiency and early diagnosis of LM.

## Related Work

### Feature Extraction and Classification

Morphological features of white blood cells (WBCs) based on traditional ML algorithms played a crucial role in the accuracy of WBC classification in recent research (18). The classifier of support vector machine (SVM) could achieve 84% accuracy of 140 digital blood smear images in five types of WBCs (19). Moreover, bi-spectral invariant features combined with the SVM and classification tree were used to 10 types of WBCs classification on three datasets of Cellavision database, ALL-IDB, and Wadsworth center and ultimately reached an averaged accuracy of 96.13% (20). Razzak and Naz (2017) extracted the features of the ELM classifier in CNN and achieved an accuracy of 98.68% in WBC classification (21).

### Deep-Learning-Based Algorithm

DL has been widely used in the WBC classification, which uses multiple processing layers to learn internal data representations from training datasets compared to traditional machine learning methods. Tiwari et al. (2018) applied data augmentation to expand training. They augmented cell images from 400 to 3,000 and achieved average precision of 88% in double convolution layer neural networks (DCLNN) (22). Convolutional neural networks (CNNs) achieved the best performance with an accuracy of 96.6% in the task of two types of WBCs classification from the ALL-IDB dataset (23). In the BCCD dataset, the implementation of recurrent neural network (RNN) in the CNN reached an accuracy of 90.79% for the task of four types of WBCs classification (24). CNN obtained an accuracy of 96.63% in five types of WBCs classification (25). The dataset proposed by Khouani et al. (2020) contains 145 labeled cells (87 images), including 49 normal cells, 24 dystrophic cells, and 72 other cells. Their study obtained 92.19% of precision by Resnet 50. It is the smallest dataset in recent research for WBC classification (26). Timely proposed CNN and RNN merging model with canonical correlation analysis illustrated an excellent performance of 95.89% to classify four types of WBCs in public data from Shenggan/BCCD data and kaggle.com/paultimothymooney/blood-cells/data (27). TWO-DCNN obtained the highest precision of 95.7%, with the most significant area under the receiver operating characteristic (ROC) curve (AUC) of 0.98 in low-resolution datasets (28).

## Materials and Methods

### Patient Cohort and Dataset

A retrospective study, which is approved by the institutional review board, was carried out from Xijing Hospital from January 2008 to December 2020. Meningeal cancer diagnosed cases through medical records followed the 2020 expert consensus (7). CSF cytology and clinical variables included demographic information, such as age, gender, etc., collected from the patients. The inclusion criteria for the meningeal carcinoma study were (i) confirmed cancer history; (ii) newly emerging neurological symptoms and clinical signs; (iii) typical CT, MRI imaging findings, or CSF cytology confirm cancer cells’ presence; and (iv) the quality of the microscope image is sufficient for analysis, without movement or artifacts. Before obtaining the optical image of the CSF cells, we strictly followed the above-mentioned diagnostic criteria, combined with the patient’s medical history, clinical signs, imaging, and cytology screening to confirm meningeal carcinoma cases.

Qualified cytologists made clinical diagnosis following clinical information, laboratory data, immunophenotyping, cytogenetic analysis, and molecular study. The inclusion criterion was that every slide of LM patients should display a cell type that belongs to one of the research’s diagnostic categories. Before the image was annotated, three trained cytologists reviewed each case’s CSF slides microscopically, including MGG staining and immunohistochemistry (IHC) for auxiliary diagnosis. In addition, the cytologists may also access patients’ medical reports if there is a need to double check. After this screening, the MGG stain qualified slides were scanned at 10 μm/pixel (100× objective) and digitized into JPG or TIF format. The slides were annotated by four medical experts, including three cytologists with 7 years of experience (examiners 1–3) and a senior medical technician with 20 years of expertise (examiner 4) based on the cytomorphologic criteria. Some of these annotations generated the ground truth for the training of CNN. In the DL models, we randomly divided the dataset into training, validation, and testing sets (**Table 1**), in which the partition was made with respect to the individual cell images rather than the patients. It is worth noting that, in pursuit of an unbiased assessment, the diagnostic annotations in all cohorts were reviewed by those four examiners *via* labelmg. The final dataset contained 53,255 cells from 90 patients. Please note that the dataset in this study cannot be downloaded directly, but it can be obtained by making reasonable requests to the corresponding author.

**Table 1:** Dataset of CNN1.

Subsets	Lymphocyte	Monocyte	Neutrophil	Erythrocytes	Cancer cell	Total
Training	8716	5360	3954	10323	8925	37278
Validation	1245	766	565	1475	1275	5326
Testing	2491	1531	1130	2949	2550	10651
SUM	12452	7657	5649	14747	12750	53255

We spent over 3 years collecting data and annotating 53,255 cells, currently the world’s most extensive clinical image dataset of cancer cells in the CSF. To the best of our knowledge, the real-world dataset we used in this research is of high quality and practically representative. Furthermore, we cooperated with around 42 doctors (with different working experience levels) to perform human screening and diagnosis to compare the proposed DL models. It highly enhanced the practicality of the experiments and the dataset, and it could benefit any future research works that rely on these types of data. Hence, the dataset in this research is considered representative and practical.

### Preprocessing and Training the CNNs

The data were preprocessed before the training process to increase the convergence speed of the algorithm. The preprocessing techniques included the reduction in input data dimensionality, noise removal, and filtering out irrelevant data. All images were in JPEG and TIF formats, with a size of 2,560 × 1,920 pixels. Annotated single cell was extracted for model development, and then, the deep CNN was trained to gain the optimum classification performance. Given the relatively moderate size of our training dataset and the limitation posed by the rarity of the disease, transfer learning strategies were used in this study. Pretrained faster-RCNN network showed excellent performance on the COCO dataset. The weights from the pre-trained Resnet-Inception-V2, excluding the top layer, were set as the pretrained state of our network and then fine-tuned for the current task (29). A separate network was trained for CNN1 (lymphocytes, monocytes, neutrophils, red blood cells, and cancer cells) and CNN2 (lung cancer cells, gastric cancer cells, breast cancer cells, and pancreatic cancer cells), resulting in two trained CNN. The optimization objective is defined as the cross-entropy between the predictions and the ground truths. In the training process, the initial learning rate is set as 0.0005, and the optimizer is Rms prop. To aggrandize data varieties (**Supplementary Tables S1**, **S2**), we performed on-the-fly data augmentations, including rotating between 0° and 30°, random flipping horizontally or vertically, randomly adjusting brightness or contrast or gamma, zooming in or out, saturation or shifting, optical or grid distortion, and elastic transformation. Finally, the optimized model with the minimum loss was saved and adopted. A good encapsulation model was used in the full images of CSF cell predictions. The networks were implemented using the TensorFlow1.14 (CUDA version 10.0.130) DL library and were trained on an Nvidia GTX 2080Ti Graphical Processing Unit machine with 11,019 Mib of VRAM.

The detection method of CNN used in this research was faster RCNN. Faster RCNN integrates feature extraction, proposal extraction, bounding box regression, and classification into one network, which dramatically improves the overall performance, especially the detection speed. Comparing RCNN and fast RCNN, faster RCNN implements Region Proposal Networks (RPN). RPN replaced the original method of using a segmentation algorithm to generate region proposals, which significantly improved the speed of detection region generation. The primary function of conv layers was to extract the feature maps of the image, which was used in the subsequent RPN layer and the fully connected layer. Conv layers contained conv, pooling, and relu three sublayers. The cell images information was the input to the network in the form of a matrix, and the matrix after the Conv layers can reflect the original picture. Each feature map sets nine candidate anchors. RPN used softmax to decide whether the anchors are positive or negative and then used bounding box regression to correct anchors to obtain accurate proposals. The feature maps and accurate proposals above were feed into the region of interest (ROI) pooling, whose output was further sent to softmax classification and bounding box regression. After the classification, the detection object category and the final precise detection frame position were obtained. In the labeling of the training set, candidate anchors are calibrated according to the different sizes of the cells. In that case, the RPN network in the neural network can be iteratively trained to well-frame the cells in the test set. ResNet-Inception-V2 network is the backbone network of faster RCNN (29). The top layer of the Resnet-Inception-V2 architecture is the softmax layer, which converts the output of the previous layer into a probability output to solve the classification problem.

### Inference and Model Evaluation

CNN1 is trained with five types of cells, including lymphocytes, monocytes, neutrophils, red blood cells, and cancer cells. This model aimed to distinguish cancer cells from four types of cells in CSF to assist in diagnosing LM. We developed a four-way cancer cell classification model to facilitate cancer target treatment, namely, CNN2, which consists of lung cancer cells, gastric cancer cells, breast cancer cells, and pancreatic cancer cells. The Xijing dataset is used in the following manner: 70% for training, 10% for validation, and 20% for testing. CNN1/2 performance was evaluated based on the overall mean average precision (mMAP) on the validation set, AUC on the test set. ROCs were plotted to show the dynamic tendency, in which sensitivity varied with specificity in the test set.

### CAD Software

CAD software was designed to transfer CNN output results to a visible CSF cytology report in the hospital (**Supplementary Figure S1**). CAD software contains the main interface, the CSF cytology input interface with patient baseline information, and the CSF cytology report interface. Trained cytologists type patients’ baseline information and input the CSF cell images, and the software would use the CNN model to detect directly. The output was transformed as a CSF cytology report interface, and the classification result for each image was stored as a CSV file in case of the double checking of cytologist consultants. Every cytology report needs a cytologist consultant signature before sending the report to the patient.

### Comparison Between the DL Model and Doctor

Multi-center tests were conducted to validate the model’s generalization abilities comprehensively. We used an additional 413 cells from the First Affiliated Hospital of Xi’an Jiaotong University and 228 cells from Tangdexu Hospital to test the accuracy between CNNs and doctors with different working experience levels. Forty-two doctors, including eight experts, 17 junior doctors, and 17 interns, were invited to review the Xi’an Jiaotong dataset and the Tangdou dataset independently and blindly. Note that the work experience of experts is more than 5 years, that of juniors is between 1 and 5 years, and that of interns is <1 year. The test was in a questionnaire, and the test time was 1 h. The task of test 1 was to classify cells into lymphocytes, monocytes, neutrophils, red blood cells, and cancer cells, while test 2 was to subclassify cancer cells into lung cancer cells, gastric cancer cells, breast cancer cells, and pancreatic cancer cells. After doctors completed the test, three of them proofread cell images again to give a standard answer. It was challenging to obtain the classification of cancer cell types in this process, which only relied on cytological features. Experts need to consider various relevant auxiliary examinations (imaging, immunohistochemistry, etc.) to make a decision. We used Cohen’s kappa coefficient to compare our deep learning model and most doctors.

### Statistical Analysis

Statistical analysis was performed using the R programming language, and the non-parametric methods were implemented using Python 3.7.

## Results

### Five-ways Cell Classification Model (CNN1)

**Figure 1:**
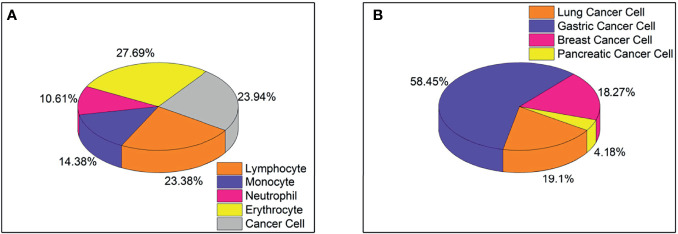
**(A)** Proportion of five types of cells, including lymphocytes, monocytes, neutrophils, red blood cells, and cancer cells, totaling 53,255 cells. **(B)** The proportion of four types of cancer cells, including lung cancer cells, gastric cancer cells, breast cancer cells, and pancreatic cancer cells, total 8499 cells.

**Figure 2:**
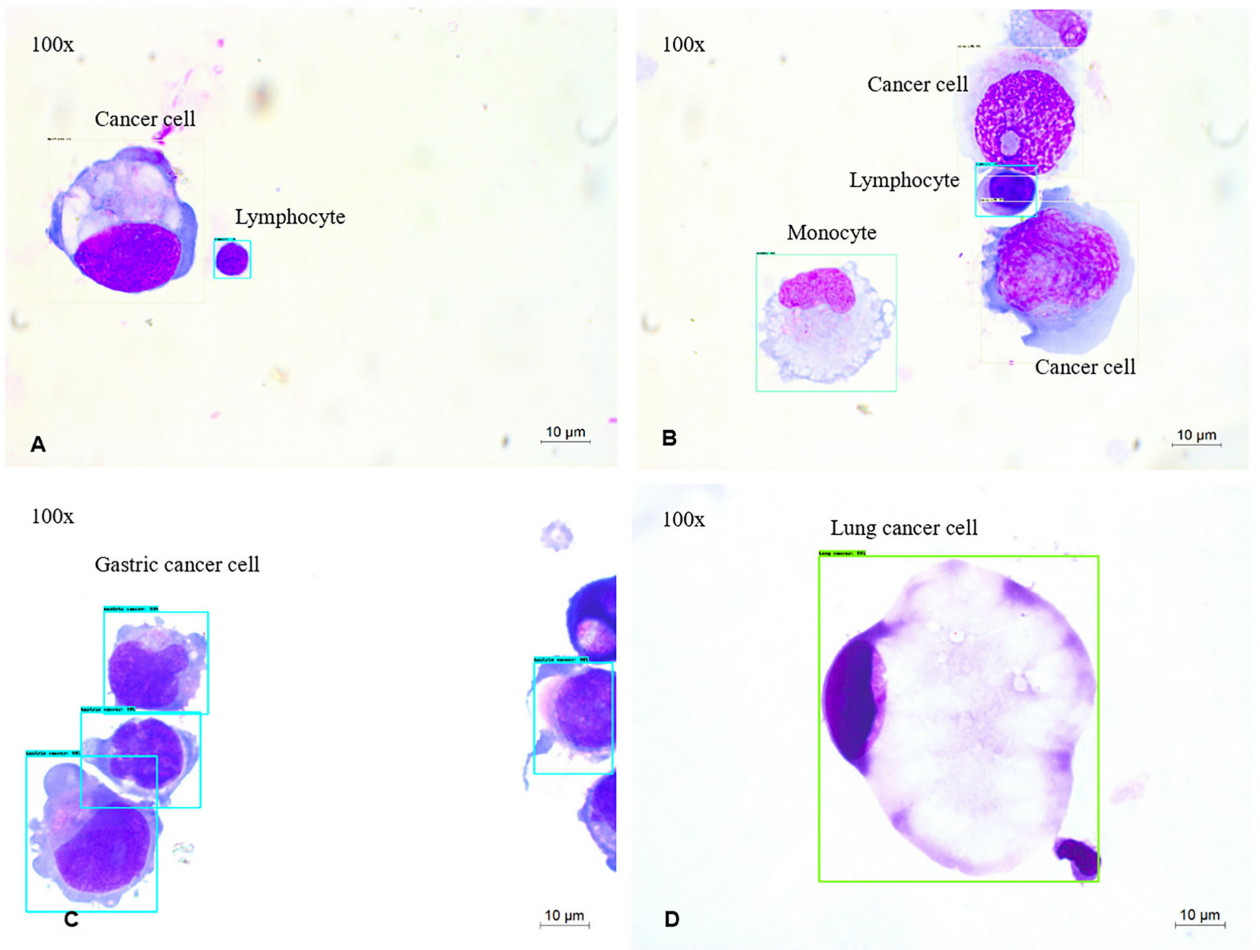
MGG Images **(A–D)** were captured with a 100× objective on a microscope **(A, B)**. The output of 4-way cell classification (CNN1) in CSF. These images were taken from slides labeled with Cancer cells (white), lymphocytes (blue), monocytes (green). **(C, D)** The output of four-way cancer cell classification (CNN2) in CSF. These images were taken from slides labeled with Gastric cancer cells (blue), Lung cancer cells (green).

The five-type classifier can distinguish cancer cells from other cells (lymphocytes, monocytes, erythrocytes, and neutrophils) in CSF, which is helpful for cytologists in the early diagnosis of LM. In this experiment, 53,255 cells were collected and labeled (**Figure 1**), including cancer cells (12,750), erythrocytes (14,747), lymphocytes (12,452), monocytes (7,657), and neutrophils (5,649). The initial dataset was randomly divided into training (n = 37,278 cells), validation (n = 5,326 cells), and test (n = 10,651 cells) subsets (**Table 1**). **Figure 2** shows the output cell image in CSF. Cytologists classify cancer cells according to cell cytology, which shows moderate pleomorphism, prominent nucleoli, and deep staining to vesicular nuclei (30).

**Figure 3:**
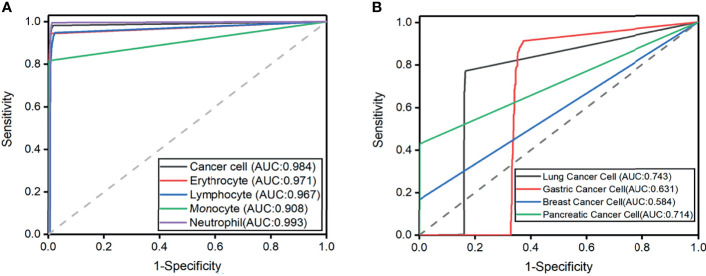
**(A)** Receiver operating characteristic curves for the five cell classification problems. Axial is 1-specificity, and the vertical axis is sensitivity. The Area under the curve (AUC) of external testing is included. **(B)** Receiver operating characteristic curves for the four cell classification problems. The Area under the curve (AUC) of external testing is included.

The highest predictive average precision (AP) in the validation set is neutrophil at 98.65%, and the lowest mean average precision (mAP) is monocyte at 91.1% (**Table 2**). In addition, the mAP for classifying types of cells is 96.15%, which is sufficient to identify cancer cells from CSF. The cohort of the test set contains 10,651 cells, and the ROC curves of each type of cell are shown in **Figure 3** with the IOU threshold set as 0.5. The AUC of cancer cells is 0.984. The proposed method demonstrates promising performance with a sensitivity of 98.21% and a specificity of 98.3%, while the rest of the cell types also have significant ROC curves. When AUC is >0.7, CNN1 is good enough to distinguish cancer cells from the other four types of cells. The rate in lymphocytes (0.967), monocytes (0.908), erythrocytes (0.993), and neutrophils (0.971) show that the model succeeds in distinguishing cancer cells from four other cell types in CSF in the external dataset.

**Table 2:** Model evaluation for CNN1.

Type	Result				
	Validation Set		Testing Set		
	*AP*	*mAP*	AUC	Sensitivity	Specificity
Lymphocyte	95.01%	96.15%	0.967	94.80%	97.63%
Monocyte	91.10%		0.908	81.70%	99.73%
Neutrophil	98.65%		0.993	99.60%	98.65%
Erythrocyte	97.93%		0.971	94.40%	99.00%
Cancer cell	98.03%		0.984	98.21%	98.30%

### Four-Ways Cancer Cell Classification Model (CNN2)

Classifying typical cancer cell types in LM by CNN2 will make it possible to predict the original cancer source. In **Figure 1**, we used a dataset of 8,499 four-class cancer cells (3,242 images) that were sub-labeled into lung cancer cells (1,623), gastric cancer cells (4,968), breast cancer cells (1,553), and pancreatic cancer cells (355). As mentioned earlier, it was difficult to classify cancer cell subtypes according to cell morphological characteristics. Hence, cytologists reviewed the patient’s medical history and auxiliary examinations to obtain an accurate cancer annotation, such as neuroimaging and immunohistochemistry. The initial dataset was randomly divided into the training (n = 5,949 cells), validation (n = 850 cells), and testing (n = 1,700 cells) subsets (**Table 3**). **Figure 2** shows the output cell image in CSF.

**Table 3:** Model evaluation for CNN2.

Subsets	Lung Cancer Cell	Gastric Cancer Cell	Breast Cancer Cell	Pancreatic Cancer Cell	Total
Training	1136	3478	1087	249	5949
Validation	162	497	155	36	850
Testing	325	994	311	71	1700
SUM	1623	4968	1553	355	8499

The experiment result showed that the mAP of lung cancer cells was the highest (80%), and the mAP of breast cancer cells was the lowest, which is 65% (**Table 4**). In addition, the mAP of cell classification was 78.00%. When the IOU threshold is 0.5, the ROC curve of each cell is shown in **Figure 3**. When the AUC of lung cancer cells is 0.718, the model achieves 78.30% sensitivity and 84.60% specificity. The possibility in the gastric cancer cell (0.631), breast cancer cell (0.584), and pancreatic cancer cell (0.714) indicated that the model succeeds in classifying four subtypes of cancer cell in CSF. It is worth noticing that the improvement of subtype cancer cells needs further study, which is considered one of our future works.

**Table 4:** Model evaluation for CNN2.

Type	Result				
	Validation Set		Testing Set		
	*AP*	*mAP*	AUC	Sensitivity	Specificity
Lung cancer cell	80.00%	79.00%	0.718	78.30%	84.60%
Gastric cancer cell	79.60%		0.606	63.90%	82.50%
Breast cancer cell	65.00%		0.584	25.70%	98.80%
Pancreatic cancer cell	78.00%		0.692	61.40%	98.90%

### Multi-Cohort Testing and Contesting With Doctors

**Figure 4:**
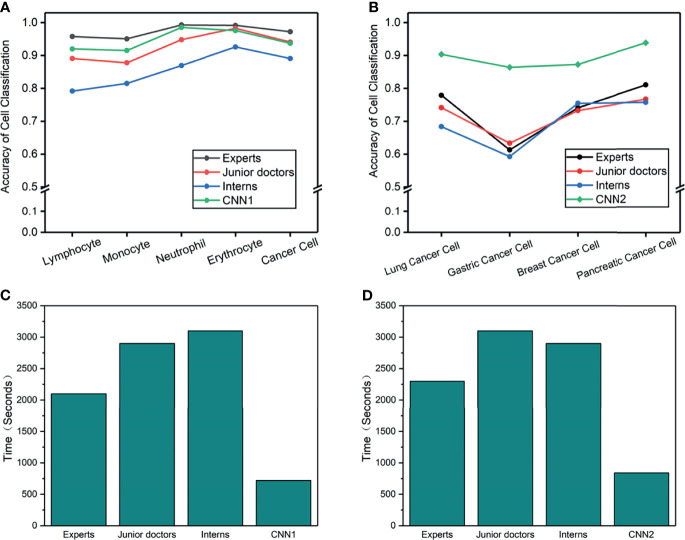
**(A–D)** Cell accuracy and time-consumed in the man-machine test. **(A)** Cell accuracy of 5-way classification in test 1. **(B) **Cancer cell accuracy of 4-way classification in test 2. **(C)** Consume time of 5-way cell classification in test 1. **(D)** Consume time of 4-way cancer cell classification in test 2.

An external validation dataset from the First Affiliated Hospital of Xi’an Jiaotong University (n = 413 cells) and Tangdou Hospital (n = 228 cells) were used in this research. Each doctor’s accuracy, specificity, and sensitivity were calculated (**Table 5**). This experiment aimed to compare the diagnosis results from the proposed model with the diagnosis results from doctors in terms of cancer cells in LM. Forty-two doctors from three different training levels (expert, juniors, and interns) were invited to test 1 and 2. The experiment results found that CNN and doctors have good performance in test 1 (the detailed results are summarized in **Supplementary Tables S3**–**S8**). Among the three doctoral groups, the accuracy of cell prediction from the expert was the highest. The average accuracy of CNN was slightly lower than that of the expert group, yet it was higher than that of the other two doctor groups. We also observed that, even if the performance of CNN1 is not the best, it can still classify cancer cells with high accuracy. Cohen’s kappa coefficient (**Table 6**) showed that the expert and CNN model strongly correlated with the standard answer, more significant than 0.86. The junior doctors had a substantial correlation with the standard answer, and the interns had a moderate correlation with the standard answer. Test 2 was more problematic, as it required sub-classifying cancer cells according to different original sites not used in CSF cytology. A surprisingly good result was that the overall accuracy of CNN2 in the classification of cancer cell subtypes was higher than that of all doctors. Meanwhile, the sensitivity and specificity of CNN2 were 10%–35% higher than that of doctors (**Table 7**). CNN2 performed better than the doctors in most of the scenarios, except that the sensitivity of breast cancer cells was relatively low. Cohen’s kappa coefficient (**Table 8**) showed that the correlation between the CNN models and the standard answer was better than all doctors. As we can see, our model accomplished comparable performance with doctors and even better in some cases. Besides, the CNN models consumed much less time in the test than doctors, who needed to speed 45–60 min in the test (**Figure 4**).

**Table 5:** Overall sensitivity and specificity in test 1.

Test 1	Sensitivity	Specificity
Experts	93.14% ± 1.90%	98.63% ± 0.37%
Junior doctors	81.20% ± 6.15%	96.26% ± 1.24%
Interns	63.01% ± 13.55%	92.60% ± 2.71%
CNN1	87.17%	97.24%

**Table 6:** Cohen’s k for test 1.

	Standard Answer			
	Experts	Junior Doctors	Interns	CNN1
Cohen’s k	0.89	0.75	0.57	0.86

**Table 7:** Overall sensitivity and specificity in test 2.

Test 2	Sensitivity	Specificity
Experts	45.61% ± 7.11%	89.13% ± 1.44%
Junior doctors	38.83% ± 7.44%	87.79% ± 1.46%
Interns	31.06% ± 5.67%	86.19% ± 1.14%
CNN2	77.63%	95.53%

**Table 8:** Cohen’s k for test 2.

	Standard answer			
	Experts	Junior doctors	Interns	CNN2
Cohen’s k	0.35	0.21	0.18	0.64

### Cytology Automatic Diagnosis Report System

**Figure 5:**
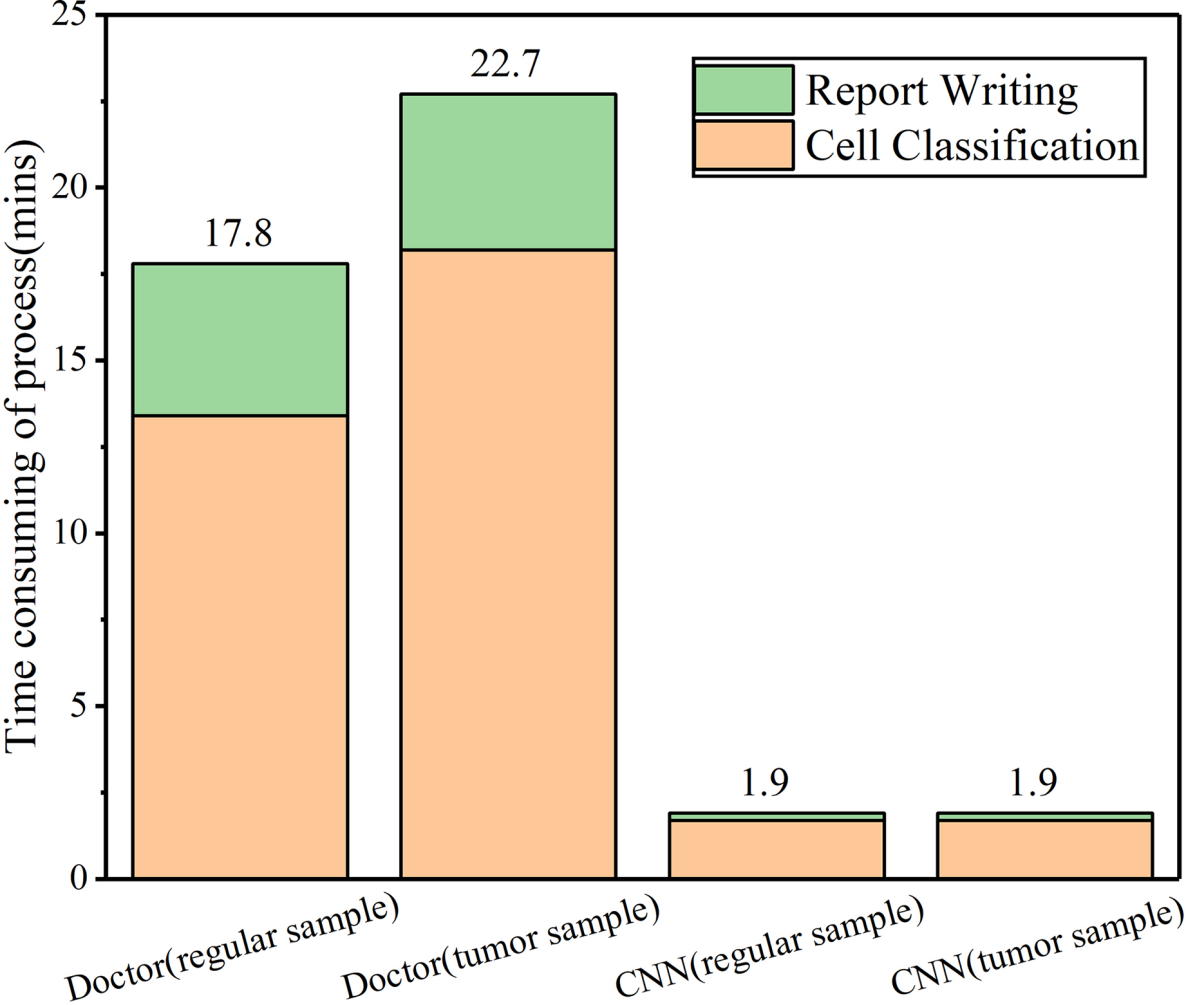
CAD and cytologists time-consuming in cell classification and report writing in CSF samples.

With the excellent performance of CNN in man-machine tests, we developed CAD software to obtain cytological diagnosis reports in this work automatically. In all our experiments, no matter how difficult the classification task was, the average cell classification report in CSF took <12 s using the CAD software, saving 96% time compared with cytologists. We constructed an automated cell classification platform based on CNN and CAD. The total processing time of cytologists ranged from 17.8 to 22.7 min, depending on different task, while CNN was relatively stable, taking 8% of cytologists (**Figure 5**). It is worth to mention that the cost of CNN combined with the CAD system platform is ~65,000 dollars; considering it has minimum 5-year service life, computerized slides expenditure is approximately 2.5 dollars per slide. As the number of computerized slides increases, the cost of each slide may be reduced to less than two dollars. The difference in cost varies with countries or regions.

## Discussion

LM, a wretched prognosis disease, is threatening many lives every year. For a long time, cytology evaluation has been the baseline for LM diagnosis, directing further examination, target treatment, and prognosis (31). Cytologists can identify cancer cells through microscopic observation to diagnose meningeal carcinoma. In addition to demographic information, such as the patient’s age and gender, CSF cytology (including size, nucleus, cytoplasm, staining matrix type, and cell distribution) is the other vital clue to help cytologists distinguish meningeal carcinoma from non-meningeal carcinoma. However, the current barrier in the medical aspect is that few cytologists can develop enough professional knowledge to make a precise diagnosis of meningeal carcinoma only from morphology to classify different cancers. Cytologists have many limitations in image recognition, and the possibility of accurately explaining the cancer source is low, which may lead to misdiagnosis and possibly harm the patient’s prognosis. Usually, it takes years for a trainee physician to become a cytology consultant.

The transition from glass slides under an optical microscope to virtual slides viewed by computers enables automatic inspection, especially with AI techniques. Medical AI is promising for improving healthcare qualities and lessening the inequality between city, urban, and rural health services. This study applies DL models and image recognition algorithms to efficiently predict lung cancer, gastric cancer, breast cancer, and pancreatic cancer cells compared with cytologists. To tackle real-world clinical problems based on MGG staining slides in CSF, we designed the five-type classifier of the cell (CNN1) for distinguishing cancer from other cells in CSF. In order to find the possible original site to facilitate the target treatment of LM, we constructed the four-type classifier of cancer cells (CNN2) to divide cancer into subtypes.

The CNN models in this study were based on Resnet-inception-V2, which achievesd a good performance in our research. The task of CNNs is not only for cell detection but also for cell location. Cell location increases the difficulty in cell detection, as most of the areas in the slides in CSF are black, with little space containing cells. Considering this situation, we used 70% of the dataset for model training and 30% dataset for validation and testing. In CNN1, the more of a cancer cell on the validation set was 95%, and the AUC of cancer cells on the testing set was 0.984. It indicated that the proposed CNN model is sufficiently good for distinguishing cancer cells from the other four types of cells in CSF. However, the mMAP of a cancer cell on the validation set was lower than 80%, and the highest AUC of cancer cell subtype on the testing set was 0.743 in CNN2. The possible reason that CNN2 showed a flat performance is because CNN2 only contained less than one-fifth dataset as CNN1 used in the research, which enlarged the scope of the dataset. Here, the clinical barrier was the shortage of LM patient samples for the known original site. Besides, the subtype of those four cancer cells belongs to adenocarcinoma, which is highly similar even if the source is different. Despite those limitations, CNN2 performance was significantly better than cytology experts in all cancer subtypes, except breast cancer. The potential reason is that breast cancer contains excessively pathology types (around 20 types) than other cell types. All the above reasons increased the analysis difficulty for CNN2 in object detection. Even so, CNN2 demonstrated that it is feasible to use AI techniques for classifying cancer cell subtypes. It is indicated that our proposed deep learning model could be adapted to more complicated situations in real clinical scenarios.

We have validated our proposed CNN models with over 50,000 cells in the Xijing dataset. We tried to interpret the differences in prediction effectiveness observed in the multi-center testing experiments and compared the accuracy between doctors with different experiences and CNNs. CNN1 had a similar accuracy with experts in terms of the Tangdou dataset. At the same time, CNN2 outperformed eight full-time cell specialists with an average increase of 10% accuracy and 15%–20% sensitivity in the cancer cell classification in terms of the Xi’an Jiaotong dataset. The possible reason that humans got the worse performance is that doctor’s diagnosis needs to consider relevant information such as medical history or primary cancer location. It is nearly impossible to directly classify the cell morphology based on human eyes without the above information in the test. Thus, it is difficult for doctors to give the specific classification without comprising cytomorphologic features in CSF with other cell types, as some atypical cancer cell is similar to lymphocyte. It is worth mentioning that our dataset contained an amount of poorly differentiated cancer cells. These cells can be classified correctly with high mAP. With the further expansion of the data volume, the CNN model can implement the classification of more types of CSF cells, and the mMAP will be further improved.

In comparison, the CNN models show their advantages in robust feature learning and image classification capabilities. The proposed CAD system consumes 90% less time processing images than doctors. LM cytology classification’s current low screening speed motivates us to apply AI to CSF cytology. However, one of the limitations of this study is that AI was used in a perfect world. A laboratory prototype that we constructed in the study is only in the phase 1 stage of the AI application blueprint. Before this medical equipment is applied to the clinic on a large scale, it needs multi-center clinical trials in the real world to verify its performance and the acquisition of marketing licenses as a product. From a laboratory prototype to a well-established product in a widely applied clinic, it takes a few years. With improved laboratory prototypes, large amount of clinician work may be replaced by AI and overcome the shortage of cytology consultants. In the future, the adoption of AI techniques in the medical system may be extended to the population for screening for meningeal carcinoma or other nerve system disease with typical cytological changes. In addition, AI techniques may assist in diagnosing or predicting cancer in an early stage, thus potentially providing more time for an effective treatment to benefit the prognosis. With technological advances, cancer type prediction and accuracy can be increased to achieve particular targeted therapy.

This research solves three fundamental difficulties: (1) obtaining large amounts of annotated training data, (2) ensuring the acquisition of equipment and modalities, and (3) persuading nearly 50 doctors with different experiences to participate in human–machine testing. First, the cell fluid center in our study is the biggest CSF center in China. This research is the world-first one that establishes a large-scale clinical MGG images dataset of meningeal carcinoma cancer cells, which is based on Xijing Hospital over several decades. We spent over three years collecting data and annotating over 50,000 cells, which are currently the world’s most extensive clinical image dataset of cancer cells in the cerebrospinal fluid. Second, considering the challenges of unclear diagnosis of meningeal carcinoma and the time-consuming process for cytology report, we not only built CNN models but also developed a CAD software, which could significantly decrease the time for cytology report. Finally, we validated our proposed CNN models by comparing them with doctors with different years’ work experience. Experiment results demonstrated that the proposed CNN models achieve a better than or sufficiently good performance as doctors.

In summary, this research demonstrates the potential of applying AI techniques to cytomorphology classification, and this research further extends to offer a CAD software to get a CSF cytomorphology report in a timely manner. However, to achieve accurate clinical utility, versatility and generalization must be adopted. It is worth noticing that these comparative experiments only show that the CNN model’s “image reading and identification” capability is better than that of doctors, but not the actual diagnostic ability to some extent.

## Conclusion

In this work, we have developed a DL model that effectively classifies the cancer cells in CSF to assist in diagnosing LM in the early stage, thanks to the use of labeled data and the step-by-step training of the CNN models. In addition, we develop a CAD system to generate cytology diagnosis reports promptly. The experiment results show that our system outperformed 42 doctors, including eight cytology experts. Our research confirms the positive influence of applying AI techniques in medical image processing. It is promising that AI could bring benefits to extend the window of cancer detection and thus potentially increase the opportunity to obtain a targeted therapy. Furthermore, the efficiency brought by the CAD software is promising to improve the healthcare quality and lessen the inequality between city, urban, and rural areas.

## Data Availability Statement

The raw data supporting the conclusions of this article will be made available by the authors, without undue reservation.

## Ethics Statement

The studies involving human participants were reviewed and approved by Xijing Hospital. The patients/participants provided their written informed consent to participate in this study. Written informed consent was obtained from the individual(s), and minor(s)’ legal guardian/next of kin, for the publication of any potentially identifiable images or data included in this article.

## Author Contributions

GaZ, WR, GN, YiG conceived the study; WJY, JWL, YSZ, JYY collected the data; WJY, YYL, YSZ, HFH, LYJ, HPW JYY preprocessed the images; TTL, HMZ, YH, TB, WD evaluated the CSF cell; XHC, JMZ, YTG, GYL, GI, RZZ, QY, YD developed the study methodology. XHC, JMZ, TMM, GI, RZZ, QY, YD, JHH, XYW, QH performed experiments in multi-cohort testing and classified the data; WJY, YSZ, DZ, JCL, HJZ, JYY, XHC, JMZ analyzed the data; GI, TMM, JHH, XYW, QH supervised the data; ZX, XFY, JHL, GXZ, DZ, YQ, MXR participated in in the analyses and discussion of the results; WJY, YYL, YSZ wrote the manuscript; GaZ, WR, GN, YiG helped in manuscript editing and revision.All authors contributed to the review, edited, and approved the final version of the manuscript. WJY, YYL, YSZ are Co-first authors, and GaZ, WR, GN, YiG are co-corresponding authors of this article.

## Funding

The project described was supported by the National Research and development precision medicine (Program 2016YFC0904500), National Science and Technology Gold Project(81671185), Natural Science Basic Research Program of Shaanxi (Program No. 2019JQ-251), Hospital-level project of Xi’an International Medical Center (Program No. 2020ZD007).

## Conflict of Interest

The authors declare that the research was conducted without any commercial or financial relationships that could be construed as a potential conflict of interest.

## Publisher’s Note

All claims expressed in this article are solely those of the authors and do not necessarily represent those of their affiliated organizations, or those of the publisher, the editors and the reviewers. Any product that may be evaluated in this article, or claim that may be made by its manufacturer, is not guaranteed or endorsed by the publisher.
