# Supplementary material for: Deep Learning-Based Classification of Cancer Cell in Leptomeningeal Metastasis on Cytomorphologic Features of Cerebrospinal Fluid
Source: Front Oncol. 2022 Feb 22;12:821594. doi: 10.3389/fonc.2022.821594 (PMC8904144; doi:10.3389/fonc.2022.821594)
Supplement: Supplementary file 1 [file DataSheet_1.doc]

### ****Supplement****


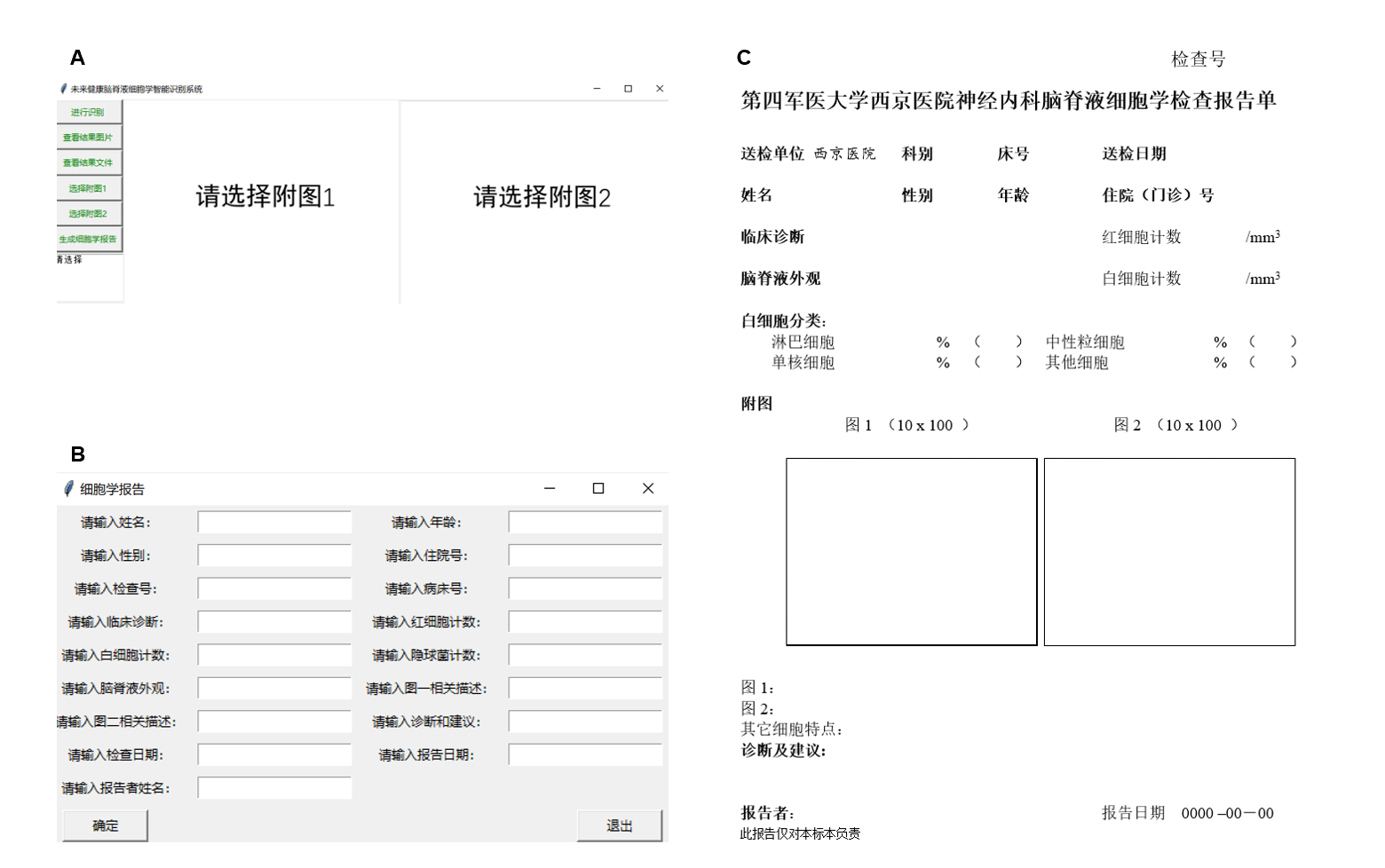


FIGURE S1 |CAD software original version in Chinese(A) The main interface of CAD software， the software's main interface, includes the function menu bar on the left, the picture display area on the right, and the execution status display area the lower left. The function menu bar includes six function buttons that the software can realize. (B) CSF cytology interface for input baseline information (C) CSF cytology report output of CAD software

TABLE S1 | CNN1 parameter in the research

| Optimizer | Rms prop optimizer |
| --- | --- |
| Learning rate | 0.001 |
| L2 regularization | 0.0005 |
| Dropout rate | 0.4/0.5 |
| Batch_size | 4 |
| IOU threshold | 0.5 |
| Weight parameters composed of Loss | first_stage_localization_loss_weight: 2.0  first_stage_objectness_loss_weight: 1.0  second_stage_localization_loss_weight: 2.0  second_stage_classification_loss_weight: 1.0 |
| Data enhancement | random_horizontal_flip  random_adjust_saturation  random_adjust_contrast  random_adjust_brightness |
| Evaluation index | coco_detection_metrics,  pascal voc detection metric |
| Loss function | Softmax loss function |

TABLE S2 | CNN2 parameter in the research

| Optimizer | Rms prop optimizer |
| --- | --- |
| Learning rate | 0.001 |
| Decay index | 0.95 |
| L2 regularization | 0.0005 |
| Dropout rate | 0.4/0.5 |
| Batch_size | 4 |
| IOU threshold | 0.5 |
| Weight parameters composed of Loss | first_stage_localization_loss_weight: 2.0  first_stage_objectness_loss_weight: 1.0  second_stage_localization_loss_weight: 2.0  second_stage_classification_loss_weight: 1.0 |
| Data enhancement | random_horizontal_flip  random_adjust_saturation  random_adjust_contrast  random_adjust_brightness |
| Evaluation index | coco_detection_metrics,  pascal voc detection metric |
| Loss function | Softmax loss function |

TABLE S3 | Accuracy of cells in test 1

| Accuracy | Experts | Junior doctors | Interns | CNN1 |
| --- | --- | --- | --- | --- |
| Lymphocyte | 95.78% ± 0.78% | 89.08% ± 3.09% | 79.18% ± 6.98% | 92.01% |
| Monocyte | 95.08% ± 1.33% | 87.79% ± 1.88% | 81.51% ± 7.10% | 91.53% |
| Neutrophils | 99.30% ± 0.39% | 94.82% ± 6.18% | 86.95% ± 9.39% | 98.55% |
| Erythrocytes | 99.17% ± 0.17% | 98.26% ± 1.63% | 92.62% ± 8.39% | 97.58% |
| Cancer cell | 97.23% ± 1.31% | 94.09% ± 1.92% | 89.09% ± 4.91% | 93.70% |
| Unknown cell | 99.76% ± 0.28% | 98.46% ± 1.20% | 96.64% ± 6.12% | 99.03% |
| Overall | 97.72% ± 0.62% | 93.75% ± 2.05% | 87.67% ± 4.52% | 95.56% |

| Sensitivity | Experts | Junior doctors | Interns | CNN1 |
| --- | --- | --- | --- | --- |
| Lymphocyte | 94.21% ± 0.79% | 75.38% ± 9.19% | 52.18% ± 21.96% | 94.29% |
| Monocyte | 87.86% ± 5.30% | 78.01% ± 14.90% | 52.58% ± 27.95% | 55.38% |
| Neutrophils | 95.41% ± 1.90% | 91.69% ± 9.23% | 83.25% ± 13.60% | 89.13% |
| Erythrocytes | 99.48% ± 1.01% | 96.73% ± 4.99% | 83.46% ± 19.46% | 94.34% |
| Cancer cell | 82.91% ± 12.18% | 63.80% ± 18.89% | 49.55% ± 18.49% | 90.38% |
| Unknown cell | 80.56% ± 30.68% | 30.88% ± 25.04% | 11.76% ± 17.40% | 100.00% |
| Overall | 93.14% ± 1.90% | 81.20% ± 6.15% | 63.01% ± 13.55% | 87.17% |

TABLE S4 | Sensitivity of cells in test 1

TABLE S5 | Specificity of cells in test 1

| Specificity | Experts | Junior doctors | Interns | CNN1 |
| --- | --- | --- | --- | --- |
| Lymphocyte | 96.58% ± 1.28% | 96.10% ± 2.45% | 93.02% ± 6.13% | 90.84% |
| Monocyte | 96.42% ± 1.85% | 89.62% ± 3.65% | 86.92% ± 11.32% | 98.28% |
| Neutrophils | 99.79% ± 0.31% | 95.21% ± 7.13% | 87.42% ± 10.43% | 99.73% |
| Erythrocytes | 99.06% ± 0.24% | 98.79% ± 0.67% | 95.78% ± 5.37% | 98.70% |
| Cancer cell | 99.29% ± 0.64% | 98.45% ± 1.64% | 94.79% ± 6.06% | 94.18% |
| Unknown cell | 99.95% ± 0.10% | 99.12% ± 1.28% | 97.47% ± 6.28% | 100.00% |
| Overall | 98.63% ± 0.37% | 96.26% ± 1.24% | 92.60% ± 2.71% | 97.24% |

TABLE S6 | Accuracy of cells in test 2

| Accuracy | Experts | Junior doctors | Interns | CNN2 |
| --- | --- | --- | --- | --- |
| Lung cancer cell | 77.91% ± 5.20% | 74.15% ± 5.81% | 68.37% ± 4.30% | 90.35% |
| Gastric cancer cell | 61.29% ± 8.85% | 63.34% ± 8.16% | 59.26% ± 6.16% | 86.40% |
| Breast cancer cell | 74.07% ± 5.04% | 73.27% ± 7.44% | 75.46% ± 4.24% | 87.28% |
| Pancreatic cancer cell | 81.03% ± 7.42% | 76.68% ± 8.92% | 75.70% ± 5.45% | 93.86% |
| Other cancer cells | 99.12% ± 0.96% | 97.21% ± 4.24% | 95.64% ± 5.39% | 100.00% |
| Non-abnormal cells | 97.86% ± 1.57% | 93.14% ± 2.66% | 87.59% ± 8.44% | 97.37% |
| Overall | 81.88% ± 2.39% | 79.63% ± 2.46% | 77.00% ± 1.89% | 92.54% |

TABLE S7 | Sensitivity of cells in test 2

| Sensitivity | Experts | Junior doctors | Interns | CNN2 |
| --- | --- | --- | --- | --- |
| Lung cancer cell | 72.32% ± 11.68% | 54.83% ± 20.96% | 46.32% ± 18.79% | 96.43% |
| Gastric cancer cell | 25.53% ± 16.94% | 24.11% ± 15.35% | 19.72% ± 9.00% | 91.55% |
| Breast cancer cell | 40.38% ± 9.66% | 34.54% ± 11.64% | 29.11% ± 12.68% | 30.77% |
| Pancreatic cancer cell | 20.49% ± 10.84% | 41.50% ± 25.07% | 31.54% ± 20.07% | 63.89% |
| Other cancer cells | NA | NA | NA | NA |
| Non-abnormal cells | 85.58% ± 12.86% | 47.29% ± 24.46% | 31.45% ± 20.62% | 88.46% |
| Overall | 45.61% ± 7.11% | 38.83% ± 7.44% | 31.06% ± 5.67% | 77.63% |

TABLE S8 | Specificity of cells in test 2

| Specificity | Experts | Junior doctors | Interns | CNN2 |
| --- | --- | --- | --- | --- |
| Lung cancer cell | 79.72% ± 4.06% | 80.44% ± 7.72% | 75.55% ± 7.68% | 88.37% |
| Gastric cancer cell | 77.47% ± 5.71% | 81.08% ± 8.77% | 77.15% ± 9.46% | 84.08% |
| Breast cancer cell | 81.02% ± 4.92% | 81.26% ± 10.56% | 85.03% ± 4.91% | 98.94% |
| Pancreatic cancer cell | 92.38% ± 7.07% | 83.27% ± 8.78% | 83.98% ± 6.14% | 99.48% |
| Other cancer cells | 99.12% ± 0.96% | 97.21% ± 4.24% | 95.64% ± 5.39% | NA |
| Non-abnormal cells | 99.44% ± 0.97% | 99.04% ± 1.08% | 94.82% ± 10.83% | 98.51% |
| Overall | 89.13% ± 1.44% | 87.79% ± 1.46% | 86.19% ± 1.14% | 95.53% |
